# Cholesterol Diet Withdrawal Leads to an Initial Plaque Instability and Subsequent Regression of Accelerated Iliac Artery Atherosclerosis in Rabbits

**DOI:** 10.1371/journal.pone.0077037

**Published:** 2013-10-17

**Authors:** Vivek Khanna, Manish Jain, Vishal Singh, Jitendra S. Kanshana, Prem Prakash, Manoj K. Barthwal, Puvvada S. R. Murthy, Madhu Dikshit

**Affiliations:** 1 Pharmacology Division, CSIR-Central Drug Research Institute, B.S. 10/1, Sector 10, Jankipuram Extension, Lucknow, Uttar Pradesh, India; 2 Toxicology Division, CSIR-Central Drug Research Institute, B.S. 10/1, Sector 10, Jankipuram Extension, Lucknow, Uttar Pradesh, India; Harvard Medical School, United States of America

## Abstract

Effect of long term cholesterol diet withdrawal on accelerated atherosclerosis in iliac artery of New Zealand White (NZW) rabbits has not been explored so far. Atherosclerosis was thus induced in rabbits by a combination of balloon injury and atherogenic diet (AD) (1% cholesterol and 6% peanut oil) feeding for 8 weeks (baseline) followed by chow diet (CD) feeding for 4, 8, 16, 32, 50 and 64 weeks. The plaque characterization was done using histology, real time RT-PCR and vasoreactivity studies. Significant elevation in plasma lipids with AD feeding was normalized following 16 weeks of CD feeding. However, baseline comparison showed advanced plaque features even after 8 weeks of CD period with significant elevation in intima/media thickness ratio and plaque area later showing reduction at 50 and 64 weeks CD periods. Lesion lipid accumulation and CD68 positivity was maintained till 16 weeks of CD feeding which significantly reduced from 32 to 64 weeks CD periods. Baseline comparison showed significant increase in ground substance, MMP-9 and significant decrease in α-actin and collagen content at 8 weeks CD period indicating features of unstable plaque. These features regressed up to 64 weeks of CD. Partial restoration of functional vasoconstriction and vasorelaxation was seen after 64 weeks of CD feeding. mRNA expression of MCP-1, VCAM-1, collagen type I and III, MMP-9, TIMP-1, IFN-γ, TNF-α, IL-10 and eNOS supported the above findings. The study thus reveals insights into initial plaque instability and subsequent regression on AD withdrawal in this model. These results are suggestive of an appropriate window for drug intervention for plaque stability/regression and restenosis as well as improves understanding of plaque regression phenomenon in this model.

## Introduction

Atherosclerosis is the predominant underlying pathology of cardiovascular disease, the most common cause of premature death in the industrialized world [1]. New Zealand White (NZW) rabbits are among commonly used animal models for atherosclerosis studies which primarily exhibits macrophage derived foam cells on saturated fat and high cholesterol diet feeding [2], [3]. Until recently, accelerated development of atherosclerosis has been achieved in various animal models by combination of balloon injury and atherogenic diet in different artery beds [3], [4]. In this context, rabbit iliac artery atherosclerosis model has been extensively employed for both atherosclerosis and restenosis studies due to varied reasons. Rabbit iliac artery shows (1) morphological similarity with human coronary artery, (2) shows propensity for uniform lesion size and distribution after balloon injury, (3) produces plaques with a smooth muscle–rich fibrous cap overlying a layer of lipid-laden macrophages and (4) has presumably less tapering effect in comparison to aorta [5], [6]. Moreover, the size similarity of stents used in human coronary artery with that of rabbit iliac artery makes it a preferable site for stent deployment [5]. Recent studies acknowledge the fact that local drug distribution in atherosclerotic artery depends upon the lesion composition and artery location [7], [8]. Hence, it becomes imperative to understand the atherosclerosis evolution and plaque features exhibited by rabbit iliac artery. Surprisingly, atherosclerotic evolution under setting of atherogenic diet withdrawal has not been studied till date in this model. Previous reports have shown that alternate cycles of high cholesterol and low or no-cholesterol diet can modify plaque characteristics and develop advanced, regressed or stable atherosclerotic plaques in rabbits [5], [9]. However, most of these basic studies did not employ balloon injury, lacked extensive plaque characterization, and used different artery sites [3], [10]–[13]. Hence, whether long term withdrawal of cholesterol feeding will produce advanced plaque, stabilization or regression of atheroma in this model remains conjectural. We now report the changes in plaque composition produced by dietary lipid lowering in rabbit iliac artery by showing (a) changes in intimal cell proliferation, (b) improvement in endothelial functionality (c) aggravation and subsequent regression of the lesion extracellular matrix and proteolytic enzymes (d) decrease in intracellular and interstitial lipid content (e) differential expression of key genes involved in atherosclerosis and (f) alteration of pathological features during regression. Results obtained provide new insight into the pathology and cellular mechanisms of plaque regression in this model, lends credence to the concept of plaque aging on cholesterol diet withdrawal and suggest drug intervention times on plaque stabilizing/regression therapies in this model.

## Materials and Methods

### Ethics Statement

Animal experimental protocols were approved by the Institutional Animal Ethical Committee, Council for Scientific and Industrial Research- Central Drug Research Institute (CSIR-CDRI) (Approval no: IAEC/2010/6). Procedures were carried out in strict accordance with the Guidelines of Committee for the Purpose of Control and Supervision of Experiments on Animals (CPCSEA) which conforms to the international norms of Indian National Science Academy (INSA). All efforts were made to improve animal welfare and minimize suffering.

### Experimental model of Atherosclerosis

Male New Zealand White rabbits with an initial body weight of 2.0–2.5 kg, maintained at Central Animal House facility of CSIR-CDRI, Lucknow were used. All animals consumed an atherogenic diet (1% cholesterol and 6% peanut oil) for 8 weeks to induce atheroma formation [14]. One week after atherogenic diet (AD) feeding, balloon induced endothelial injury in iliac artery was performed through a saphenous artery cutdown as described previously [15], [16]. Briefly, induction anesthesia was performed by concurrent intramuscular injection of ketamine (35 mg/kg) and xylazine (5 mg/kg) respectively and maintained with 1% to 3% isoflurane delivered in oxygen (2.0 l/min) (Harvard Apparatus Inc., USA). To prevent thrombus formation, heparin 100 U/kg was intravenously administered prior to the intervention studies. Under aseptic conditions, a 2F Fogarty balloon embolectomy catheter (Edwards Life Sciences, USA) was inserted retrograde into the iliac artery. The balloon was inflated with normal saline and pulled back three times through the iliac arteries [16]–[18]. The same investigator (V.K.) performed all of the balloon injuries. At the end of the study in respective groups, the animals sacrificed as per IAEC guidelines. To minimize the effect of anatomical tapering, the iliac arteries of each animal were cross-sectioned into three segments: proximal segment (near aorta-iliac bifurcation) was formalin fixed for histology, middle for mRNA analysis and distal for vasoreactivity studies [19].

### Experimental Protocol

**Figure 1:**
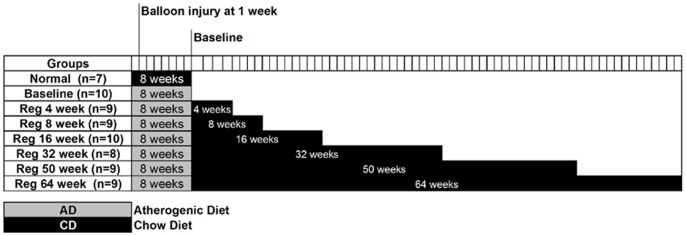
Experimental Protocol. Sixty four male New Zealand White rabbits were fed atherogenic diet (AD) for 8 weeks to create atheroma. Seven animals were fed chow diet (CD) for 8 weeks. Balloon injury of iliac artery by Fogarty embolectomy catheter was performed 1 week after initiation of atherogenic diet. Baseline group was set at 8 weeks of atherogenic diet feedingfollowed by chow diet (CD) from 4 weeks up to 64 weeks in respective groups. n = number of animals

Male New Zealand White rabbits were supplemented with *ad libitum* chow diet (CD) and atherogenic diet (AD) for 8 weeks respectively. Animals of the normal group (n = 7) received CD for 8 weeks and were immediately sacrificed thereafter. At the end of 8 weeks period of AD feeding, sixty four animals were divided into seven groups on the basis of similar ranges of hypercholesterolemia. Baseline group (Baseline; n = 10) animals were sacrificed immediately after the 8 weeks of AD feeding, while other groups were sacrificed after AD cessation and CD resumption for 4 weeks (Reg 4 week; n = 9), 8 weeks (Reg 8 week; n = 9), 16 weeks (Reg 16 week; n = 10), 32 weeks (Reg 32 week; n = 8), 50 weeks (Reg 50 week; n = 9) and 64 weeks (Reg 64 week; n = 9) respectively (Figure 1).

### Plasma lipid analysis

The blood was withdrawn from central ear artery in a tube containing 3.8% tri-sodium citrate. Whole blood was centrifuged at 4000 g for 10 min at 4°C to obtain plasma. Total cholesterol (TC), triglycerides (TG), high density lipoprotein cholesterol (HDL-C) and low density lipoprotein cholesterol (LDL-C) were estimated using Automated analyzer (Beckman Coulter Inc., USA) using commercial kits [20].

### Histology

At termination, iliac artery portion near to aorta-iliac bifurcation was isolated and preserved in 10% buffered formalin solution for 24 hours. One proximal portion of the artery was frozen in OCT compound for cryostat sectioning and other portion was processed for paraffin embedding. Arteries of all groups underwent simultaneous and similar fixing, dehydration and wax infiltration procedures to nullify the dimension changes induced by solvent treatments. Serial paraffin sections (5 µm) were collected throughout arterial length for staining procedures. For general architecture of plaque, Hematoxylin and Eosin (HE) staining and for cellular components Movat Pentachrome staining wherein muscle is stained as red, fibrinoid tissueshows as intense red, mucin/ground substance is stained blue to blue green, black color reflects nuclei and elastic fibres and yellow color depicts collagen and reticular fibres[21]. Picrosirius red staining was used to identify the type of collagen fibers (type I: yellow/orange, and type III: green, under polarized light). The sections were photographed with the same exposure time for each section [22], [23]. Lesion lipids were visualized on frozen sections using oil red O staining.

### Morphometric analysis

Structural changes in lesions of each group of animals were assessed in consecutive sections stained with Movat Pentachrome. The following features were employed for describing general lesion morphology of respective groups: i) internal elastic lamina (IEL) rupture (intimomedial change), ii) media atrophy, iii) media breakdown, iv) adventitial breakdown and v) inflammation [4], [24]. Rupture of the IEL was identified as a fracture of the IEL resulting in contact between the intima and media [4], [24]. Since the media of the iliac artery of non disease rabbits usually consisted of 7±0.4 lamellar units, medial atrophy in disease rabbits was defined as the loss of more than 4 units and/or lamellar collapse as previously described [4], [24]. Medial breakdown was identified when the elastic lamellae appeared fragmented and intracellular or extracellular lipid deposits were present [4], [24]. Adventitial breakdown was assigned when the overlying media was destroyed and lipid infiltration progressed into the adventitia. Inflammation was assigned when at least 25 or more round mononuclear cells were present within a field viewed with ×40 magnification [25]. In a pilot experiment, sections from each 300 µm interval along the length of the vessel were analyzed; these data were then averaged. As a comparison, the data sets were also analyzed using the section with the greatest neointima (data not shown). Results of this subset were within 5% of the mean when multiple sections were analyzed. These data indicate that the injury along the rabbit iliac artery is relatively uniform within the region examined. Intimal thickness (µm), medial thickness (µm), lumen area (mm^2^), plaque area (PA) (mm^2^) and percentage cross sectional narrowing (%CSN) was carried out in at least 25–30 sections throughout the arterial segment by blinded investigators employing computer-assisted image analysis software on Movat Pentachrome-stained arterial sections (Leica Qwin version 3.5.1, Heerbrug, Switzerland). Intra-observer variation was less than 5%. The intimal thickness was measured by drawing lines perpendicular to the lumen at five different locations of a stained section and the mean value was calculated [4]. Similar procedure was carried out for measurement of medial thickness. Intimal medial thickness ratio (IMT) was calculated. At the lesion site, the area of the lumen without plaque (lumen area) and the area circumscribed by the internal elastic lamina, IEL (the potential lumen area in the absence of atherosclerosis) were traced on stained sections. The plaque area (PA) was calculated by subtracting the lumen area from the IEL area. However, in plaques with discontinued IEL, the portion of the plaque that invaded the media was also included in the calculation of the PA. The cross-sectional narrowing (CSN) was defined as the extent to which the PA occupied the potential lumen area and was calculated as %CSN =  (PA/IEL area) ×100 [4]. The area of positive staining of alcian blue (an individual component of movat pentachrome staining which stains ground substance), α-actin, RAM-11 and metalloproteinase-9 (MMP-9) was expressed as a percentage of stained area divided by the plaque area of the iliac artery in at least 20–30 high-power fields (20× magnification) of sections of respective group animals throughout the artery length [26], [27]. The lipid composition of the lesion was determined by calculating the percent of the oil red O positive area to the total cross-sectional vessel wall area [28]. For polarized light images and 5× images we used a Leica DM5000B microscope. For rest of images we used a Leica DM6000B microscope. Leica application suite (LAS) software package version 3.1.0 was used for processing and saving images. The measurements taken were calibrated by selecting a calibration factor appropriate to the objective lens used in respective microscopes.

### Immunohistochemistry

Paraffin-embedded consecutive sections were stained with the following antibodies: Mouse monoclonal anti rabbit CD68 (RAM11, Dako, USA), alpha smooth muscle actin (1A4, Sigma, USA) and MMP-9 (56-2A4, Calbiochem, USA). Immunoreactivity was revealed using the Novacastra Novolink Polymer detection system (Leica Microsystems, USA) and counterstained with hematoxylin. Appropriate positive control was used for all antibodies. Negative controls included omission of the primary antibody and use of non-immune mouse IgG (Santa Cruz Biotechnology, USA) or secondary antibody only. In all cases, negative controls showed insignificant staining.

### Assessment of endothelial function

4 mm long iliac artery segments were mounted in a 10 ml organ bath containing Krebs solution, maintained at 37°C and continuously oxygenated with 95% O_2_–5% CO_2_. The tissue was kept under a constant tension of 2 g throughout the experiment and was equilibrated by intermittent changing of Kreb's buffer for 90 min [29]. Isometric measurements were recorded with force transducers (FSG-01, Budapest, Hungary) using S.P.E.L. solution Pack for Experimental Laboratories ADVANCE ISOSYS data acquisition and analysis software. Iliac artery segments were pre-constricted with incremental doses of phenylephrine (PE) (1 nM–100 µM). The extent of vasoconstriction was expressed as gram tension. After pre-constriction to 50% of maximal response of phenylephrine, rings were exposed to incremental doses of acetylcholine and relaxation curves in response to acetylcholine (Ach) (3 nM to 3 mM) were generated [30], [31]. The extent of vasorelaxation was expressed as gram tension on phenylephrine pre-contracted rings [29], [32].

### Real time RT-PCR

Quantitative gene expression analysis was performed by using SYBR Green technology. Total RNA was extracted from freshly isolated injured iliac artery of different groups using TRIZOL isolation procedure as described previously [33] and cDNA was synthesized using RevertAid™ H Minus first strand cDNA synthesis kit as per manufacturer's protocol (Invitrogen, USA). mRNA expression of key genes involved in atherosclerosis was quantified using specific primers (Table S1). Real-time RT-PCR was carried out in LightCycler® 480II Real-Time PCR system from Roche applied science (Indianapolis, USA). Amplification conditions were used in this study consisted of an initial pre-incubation at 94°C or 95°C for 10 min followed by amplification of the target DNA for 45 cycles [95°C for 10 s and 54–60°C (as applicable) for 10 s]. Melting curve analysis was performed immediately after amplification using manufacturer's protocol. Relative mRNA expression was calculated by using comparative cycle threshold (2−^ΔΔ^ *C*t) method using GAPDH as an internal standard [34].

### Statistical analysis

The results were calculated as mean ± SEM. The statistical significance of difference between different groups was determined by one way ANOVA followed by Dunett's post-hoc test using GraphPad Prism 5 software. P value of 0.05 or less was chosen for statistical significance.

## Results

### Blood lipid levels

The baseline group showed significant increase in TC, TG, LDL-C and HDL-C (p<0.001) compared to normal animals. During regression period, plasma cholesterol levels dropped significantly in Reg 4 week (455.9±33.7; p<0.001 *vs* baseline) and Reg 8 week (323.3±37.1; p<0.001 *vs* baseline) groups respectively, although it was still elevated in comparison to normal group. Aged animals till 5 years of age fed chow diet do not exhibit any significant changes in plasma lipid levels in comparison to young rabbits [35]. Similarly, TG and LDL-C levels dropped progressively and returned to normal levels at 8 weeks and 16 weeks respectively after AD withdrawal (Table 1). Elevated plasma HDL levels in baseline group reverted back to normal after 4 weeks of AD withdrawal (Table 1).

**Table 1:** Lipid profile and histological measurements of iliac artery in various experimental groups.

Parameters (mg/dl)	Normal	Baseline	Reg 4 week	Reg 8 week	Reg 16 week	Reg 32 week	Reg 50 week	Reg 64 week
TC	59.0±5.8	1484±33.8 ^***^	455.9±33.7^###^	323.3±37.1^###^	53.1±3.9^###^	40.5±3.9^###^	43.9±4.6^###^	49.6±4.6^###^
TG	66.1±4.3	356.5±65.7^***^	189.1±8.5^###^	71.7±7.5^###^	69.3±7.4^###^	57.7±8.4^###^	54±6.1^###^	56.7±7.1^###^
LDL	9.3±0.8	320.5±22.3^***^	266.1±22.9	189.7±24.2^###^	20.8±1.8^###^	10.6±0.6^###^	9.6±0.7^###^	10.1±0.8^###^
HDL	12.4±0.4	22.8±1.0^***^	14.1±1.3^###^	10.4±1.6^###^	11.1±0.3^###^	10.7±0.5^###^	11.3±0.5^###^	8.5±0.4^###^
I/M thickness ratio	0.00±0.00	3.7±0.30^***^	6±0.53	10.3±0.60^###^	6.7±0.52^###^	3.5±0.35	3.1±0.26	1.8±0.15^##^
Plaque area (mm^2^)	0.00±0.00	0.75±0.06^***^	0.86±0.05	1.5±0.1^###^	0.9±0.09^###^	0.54±0.04	0.47±0.05^#^	0.38±0.05^##^
Lumen area (mm^2^)	0.45±0.02	0.09±0.01^***^	0.12±0.02	0.15±0.01	0.1±0.01	0.20±0.02^###^	0.24±0.02^###^	0.23±0.02^###^
% CSN	0.00±0.00	81.3±1.4^***^	86.7±2.8	85.5±1.6	73.6±1.7	56.4±1.2^###^	56.3±4.2^###^	54.3±8.3^##^

Results expressed as mean ± SEM. TC: Total Cholesterol, TG: Triglyceride, LDL: Low density lipoprotein, HDL: High density lipoprotein, I/M thickness ratio: Intimal/medial thickness ratio, %CSN: Percentage cross sectional narrowing. ***p<0.001 vs. normal; #p<0.05, ##p<0.01 and ###p<0.001 *vs* Baseline. number of sections = 25–30 sections/group.

### Cholesterol withdrawal leads to an initial increase followed by progressive decrease in intima-media thickness (IMT) ratio, plaque area, lesion lipid and macrophage foam cell content

**Figure 2:**
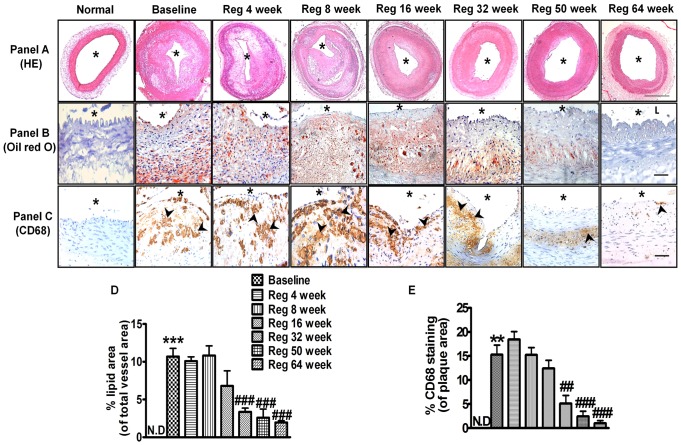
Regression phase leads to progressive decrease in percentage lipid area and macrophage foam cell content. Panel (A) Representative images of hematoxylin and eosin stained sections of all groups (Scale bar  = 500 µm). Panel (B) Representative images of Oil Red O stained cryostat sections of all groups (Scale bar  =  50 µm). Panel (C) Immunohistochemical staining showing CD68 positive cells in respective groups. (Scale bar  = 50 µm) (D) Quantitative analysis of percentage lipid area (lumen area included) in respective groups (E) Quantitative analysis of CD68 positive labelling in respective groups. **p<0.01 and ***p<0.001 *vs* normal; ^##^p<0.01 and ^###^p<0.001 *vs* Baseline. (* indicates lumen, arrow heads indicate positive staining)

Arteries in normal group, uninjured arteries and Reg 64 week group showed no intimal thickening (data not shown) with no incidence of foam cell formation. In baseline group, atherosclerotic lesion showed significant increase in arterial IMT ratio, plaque area and % CSN (p<0.001) and significant decrease in lumen area (p<0.001) compared to normal animals (Table 1). The baseline group showed extensive lipid infiltration and macrophage derived foam cell formation. Significant increase in percentage of lipid area (p<0.001 *vs* normal) and CD68 (p<0.01 *vs* normal) positive cells was observed both in the neointimal area towards lumen as well as towards IEL. Interestingly, compared to baseline group, IMT ratio and plaque area was significantly increased (p<0.001) at Reg 8 week group signifying enhanced plaque burden even after 8 weeks of AD withdrawal. The percentage lipid area and CD68 positive area was also maintained up to the baseline group levels in this group, which was progressively decreased in late regression groups (Reg 32 week to Reg 64 week) (Figure 2). The results gave a first line indication that plaque development is still occurring even after 8 weeks of AD withdrawal. Other groups in regression phase (Reg 32 week to Reg 64 week) showed progressive decrease in IMT ratio, plaque area and % CSN depicting progressive reduction in plaque burden up to 64 weeks of AD withdrawal (p<0.01 *vs* baseline at Reg 64 week) (Table 1). Concomitantly, a significant increase in lumen area was observed in Reg 32 week, Reg 50 week and Reg 64 week groups (p<0.001 *vs* baseline) respectively along with corresponding decrease in percentage cross sectional narrowing (p<0.01 *vs* baseline) (Table 1). These results indicated a brief period of plaque development even after AD withdrawal, with subsequent regression over a long period of CD.

### Biphasic response of ground substance and matrix metalloproteinase-9 during atherogenic diet withdrawal

**Figure 3:**
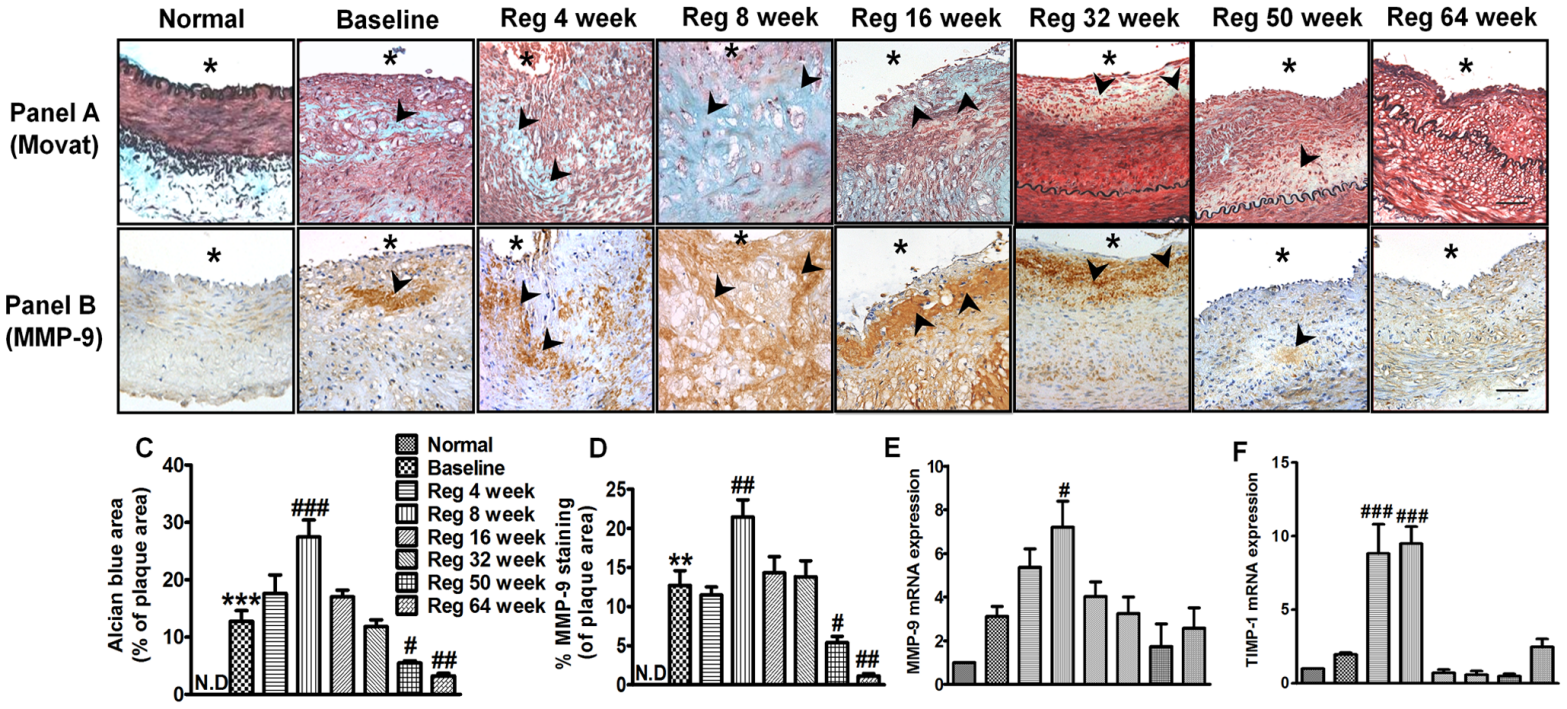
Biphasic elevation and subsequent regression of ground substance and matrix metalloproteinase-9. Panel (A) Representative images of movat pentachrome stained sections of all groups (Scale bar  = 50 µm). Panel (B) Immunohistochemical staining showing MMP-9 positive area of all groups (Scale bar  = 50 µm). (C) Quantitative analysis of alcian blue stained area in respective groups (D) Quantitative analysis of MMP-9 positive areas in respective groups (E) Arterial MMP-9 mRNA expression (fold change over normal) in all groups as determined by real time PCR (F) Arterial TIMP-1 mRNA expression (fold change over normal) in all groups as determined by real time PCR. **p<0.01 and ***p<0.001 *vs* normal; ^#^p<0.05, ^##^p<0.01 and ^###^p<0.001 *vs* Baseline. (* indicates lumen, arrow heads indicate positive staining)

General nature of extracellular matrix (ECM) and its evolution was also ascertained using Movat Pentachrome and MMP-9 staining in serial sections in respective groups. Ground substance is composed of extracellular matrix components including proteoglycans and other matrix substances including MMPs in a proenzyme form responsible for lipid entrapment and plaque instability [36]. The pictorial representations shows MMP-9 staining in similar areas of alcian blue staining in movat pentachrome stained sections (Figure 3 Panel A and B) thus validating the aforementioned fact. Baseline group exhibited a significant increase in alcian blue positive area (p<0.001 *vs* normal) thus depicting enhanced ground substance accumulation (Figure 3C). MMP-9 mRNA expression (3 fold) and protein expression (p<0.01 *vs* normal) was increased in baseline group (Figure 3D and E). Compared to the baseline group, ground substance (p<0.001), MMP-9 mRNA (3 fold increase, p<0.05) and protein expression (p<0.01) increased significantly in Reg 8 week group, thus implicating a tendency for more complex plaque formation leading towards plaque instability at this time point. Concomitant increase in TIMP-1 (a tissue inhibitor of metalloproteinase) expression, was noted in Reg 4 week (8 fold, p<0.001) and Reg 8 week group (9 fold, p<0.001) (Figure 3F) with subsequent reversion to normal in other groups. Other regression groups (Reg 50 week and Reg 64 week) showed progressive reduction in proteoglycan content (p<0.01 *vs* baseline) and MMP-9 protein (p<0.01 *vs* baseline) and its mRNA expression pattern demonstrating a tendency for plaque stabilization (Figure 3C, D and E). The results indicate a biphasic evolution of ECM production with initial plaque instability and subsequent regression on AD withdrawal.

### Assessment of plaque collagen (sirius red staining) and α-actin smooth muscle content in various groups

**Figure 4:**
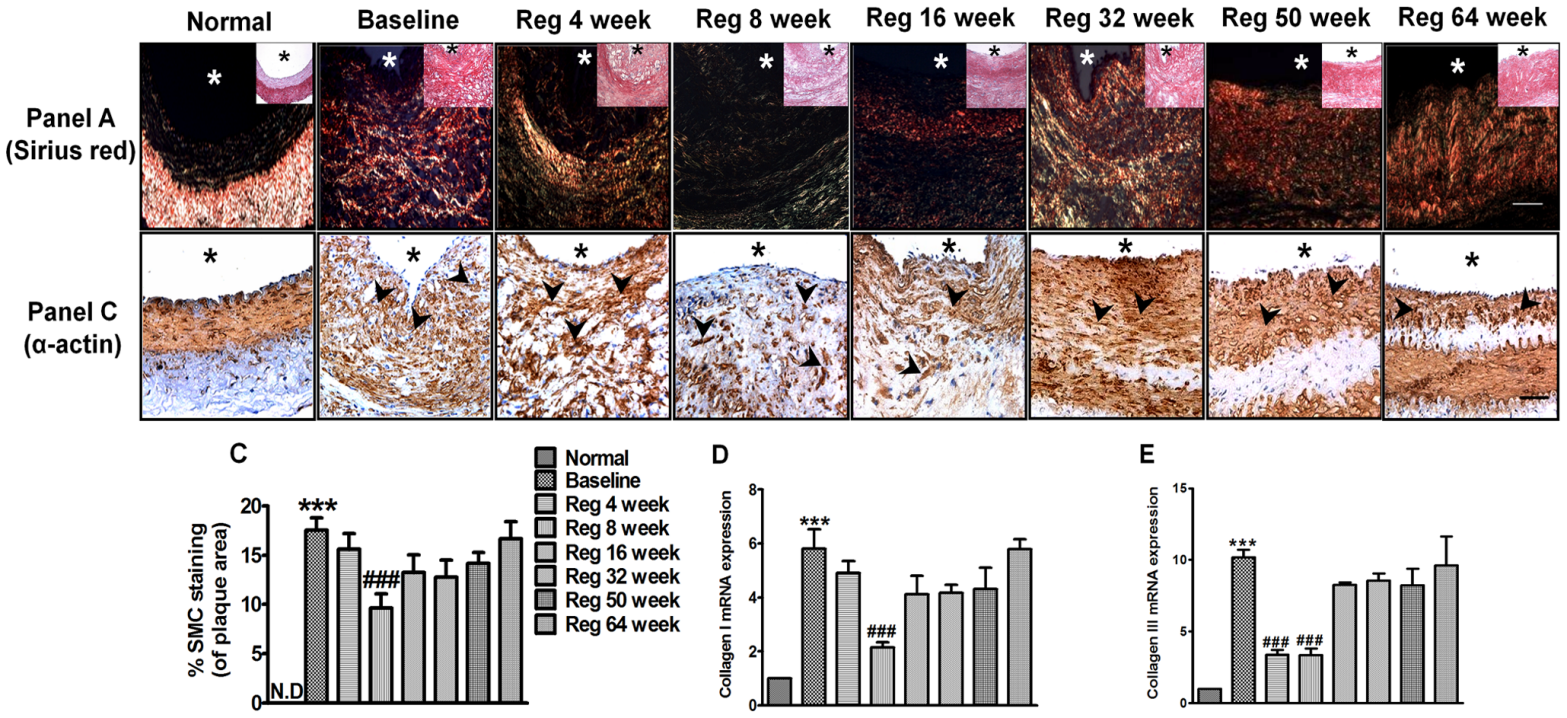
Collagen (Sirius red staining) and α-actin smooth muscle content in various groups. Panel (A) Representative images of Siruis red stained section of all groups under polarized light. Brightfield image of same section is shown as an inset (Scale bar = 50 µm). Panel (B) Immunohistochemical staining of α-actin in sections of all groups (Scale bar = 50 µm). (C) Quantitative analysis of α-actin positive stained area in respective groups (D) Arterial collagen I mRNA expression (fold change over normal) in all groups as determined by real time PCR (E) Arterial collagen III mRNA expression (fold change over normal) in all groups as determined by real time PCR. ***p<0.001 *vs* normal; ^###^p<0.001 *vs* Baseline. N.D =  not detectable (* indicates lumen, arrow heads indicate positive staining)

Plaque collagen and smooth muscle content are known to provide stability to atheromatous lesions and prevent their rupture [37], [38]. Sirius red staining showed rich presence of type I collagen in baseline group lesion area. However, normal artery staining shows only type III collagen in medial layer (Figure 4 Panel A). Significant increase in mRNA expression of both collagen I (6 fold) and III (10 fold) was observed in baseline group (Figure 4D and Figure 4E respectively). The α-actin content was significantly increased in baseline group (p<0.001 *vs* normal) hence depicting a fibromuscular hyperplasia response (Figure 4 Panel B & Figure 4C). Concomitantly, significant decrease in both α-actin (p<0.01 *vs* baseline) (Figure 4C) and collagen content was observed in Reg 8 week group (Figure 4D and Figure 4E). Cumulating the above findings, animals at 8 weeks after AD withdrawal showed some features of human unstable plaque. Subsequently, the plaque collagen content and α-actin area occupying the plaque area also progressively increased from Reg 16 weeks to Reg 64 week group indicating increase in fibrous content (collagen) and restoration of tissue integrity (SMCs) thus depicting tendency towards plaque stabilization.

### General morphological summary of atherosclerotic plaques

The atherosclerotic plaque in baseline group was presented as fibrocellular hyperplasia. In baseline group, the lesion prominently composed of foam cells occupying on an average 10% to 15% of the plaque area, with the remainder of the plaque area being occupied by smooth muscle cells and fibrous tissue matrix. There was general incidence of IEL duplication at the site of IEL breakage (Figure S1A) and medial elastic fibres duplication (Figure S1B) in all groups. This seems to be an arterial backup mechanism to protect the integrity of the artery and is derived from collagen [39]. In baseline group, there was incidence of focal medial breakdown with IEL breakdown (Figure S1C). At some places in baseline group medial fibrotic reaction was occurring with medial elastic lamellae becoming prominent (Figure S1D). The lipid core in this model (baseline group) was not typical of combination of cholesterol clefts and lipid gruel but mainly composed of extracellular lipid and foam cell deposition (Figure S1E). There was presence of smooth muscle cells overlying macrophage accumulation which was reminiscent of the structure of human atherosclerotic plaques. However, an important difference was the absence of plaque rupture and thrombosis in any of the groups. There was no incidence of fibrous cap or fibrin deposition in any of the groups. Plaques of baseline group animals exhibited features of human type IV lesion with respect to lipid core with fibrous tissue formation. One striking feature of this model is that on removal of AD for 8 weeks (Reg 8 week group) the plaque reached more complexity with abundant infiltration of foam cells into the media, leading to medial breakdown (Figure S1F). Animals in this group (Reg 8 week) exhibited complete medial destruction which did not progress towards EEL breakdown (Figure S1F). The medial foam cells were intermingled with fibrous tissue and smooth muscle cells through the defect in the internal elastic lamina in this group. At some places in Reg 8 week group there was incidence of focal medial compression extending towards atrophy (Figure S1G, Reg 8 week) and increased medial atrophy (Figure S1H). The intimal lesion was rich in ground substance with rich foam cell infiltration and less collagen content in this group. There was presence of inflammatory cells in intimal lesion and in medial layer but did not sufficiently qualify under histological definition of inflammation (25 or more mononuclear cells under 40× magnification). Subsequent withdrawal of AD in Reg 16 week through Reg 64 week resulted in progressive decrease in sub-endothelial macrophage foam cell layer (Figure 2 Panel C). The characteristic calcification response was absent in any of the groups. The general features of Reg 64 week group showed disappearance of MMP-9 and reduction in intimal cell proliferation (Figure 3 and Table 1). The medial layer recovered to a large extent in this group. In spite of IEL breakage at some places there was no damage to SMCs in medial layer (Figure S1I, Reg 64 week). There was a focal presence of cholesterol clefts in some animals of late regression groups (Reg 50 week and Reg 64 week) (Figure S1J). The lesions in all groups lacked necrotic core formation.

### Plaque regression leads to progressive increase in vasoconstriction and vasorelaxation responses

**Figure 5:**
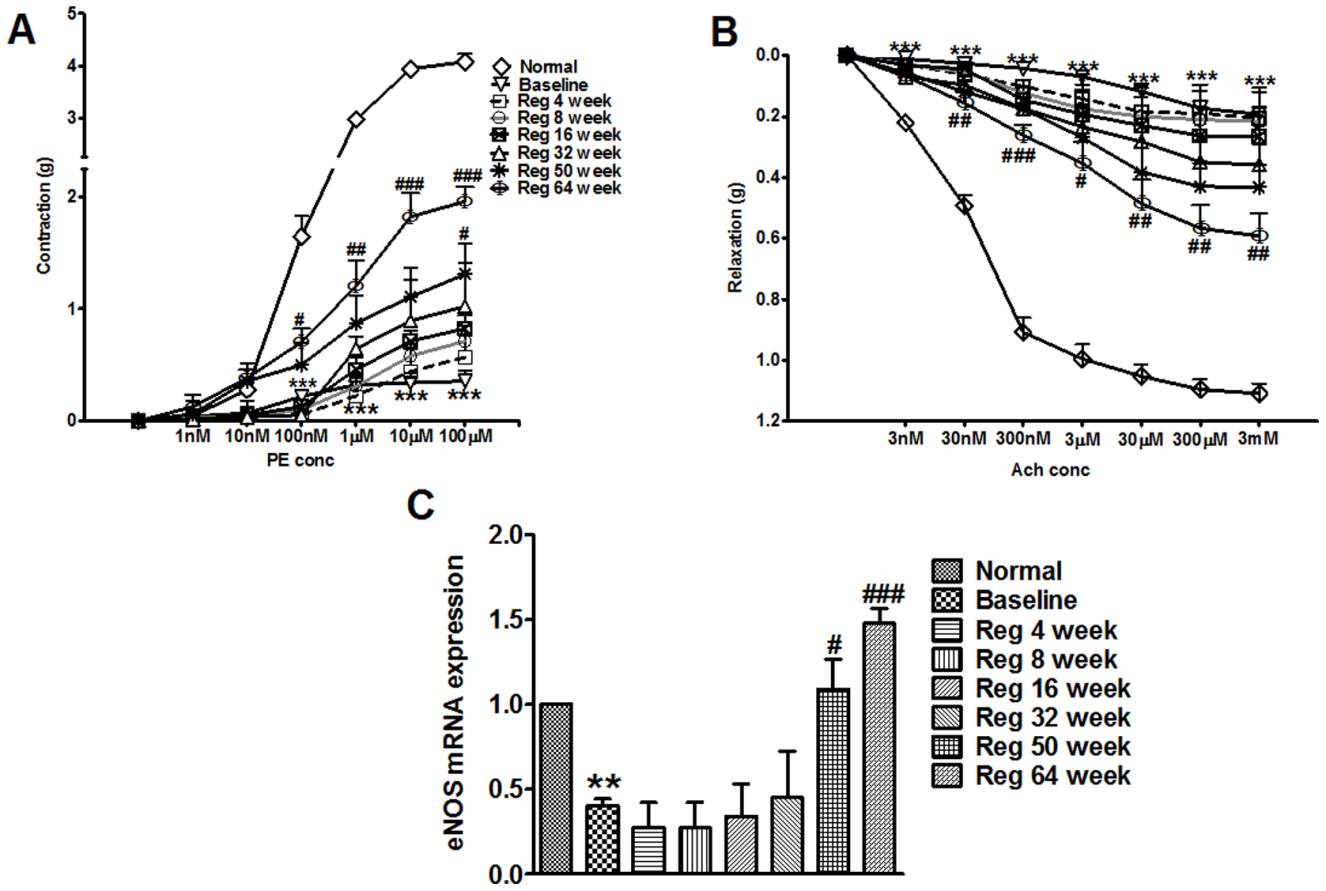
Vasoreactivity studies of injured iliac artery of all groups. (A) Effect of incremental doses (1 nM–100 µM) of phenylephrine induced contractions on atherosclerotic iliac arteries of respective groups (B) Effect of incremental doses (3 nM–3 mM) of acetylcholine induced relaxation on atherosclerotic iliac arteries of respective groups (C) Arterial eNOS mRNA expression in all groups as determined by real time PCR. **p<0.01 and ***p<0.001 *vs* normal; ^#^p<0.05, ^##^p<0.01 and ^###^p<0.001 *vs* Baseline.

The injured arteries were subjected to PE-mediated contractions and Ach induced relaxation responses to check restoration of the endothelium integrity, a prominent feature during regression phase. The combination of balloon injury and hypercholesterolemia in baseline group arteries showed significant inhibition in PE mediated contraction and Ach mediated relaxation responses. The maximal response to exogenous phenylephrine was significantly reduced in the injured vessels of baseline group (p<0.001 *vs* normal) (Figure 5A). The vascular responses to phenylephrine were almost irreversible up to the Reg 32 week group. However, in Reg 64 week the arteries showed significant elevation in PE induced contraction (p<0.001 *vs* baseline) (Figure 5A). Likewise, in comparison to normal arteries, maximal relaxation induced by acetylcholine was impaired in baseline group injured arteries (p<0.001 *vs* normal) along with significant decrease in eNOS mRNA expression (p<0.001 *vs* normal) (Figure 5B and Figure 5C). However, injured arteries in Reg 64 week group showed a significant increase in vasorelaxation responses (p<0.05 *vs* baseline), signifying revival of functional endothelium (Figure 5B). This effect was coupled with enhanced eNOS expression in Reg 50 week (p<0.05) and Reg 64 week groups (p<0.001) compared to baseline group (Figure 5C).

### Status of adhesion molecules and inflammatory cytokines during regression phase

To explore possible cause or effect of plaque regression on key genes involved in atherosclerosis, we did real time mRNA expression of atherosclerotic iliac arteries. The baseline group showed enhanced mRNA expression of VCAM-1 (4 fold), MCP-1 (4 fold), TNF-α (4 fold), TGF-β1 (10 fold) in comparison to normal (Figure S2A, S2B, S2C and S2E). However, IFN-γ expression in baseline group showed an increasing trend which was not significant. Conversely, anti-inflammatory cytokine IL-10 expression showed significant decrease in baseline group (4 fold) which was significantly elevated on baseline comparison in Reg 16 week, Reg 32 week, Reg 50 week and Reg 64 week groups thus participating in amelioration of inflammatory flux during plaque regression (Figure S2F). Previous results showed that Reg 8 week group showed maximum plaque complexity in this model. Buttressing the above results at transcription level, this group showed significant increase in MCP-1 (7 fold), TNF-α (8.6 fold), IFN-γ (4.3 fold) and TGF-β1 (26 fold) which progressively decreased as plaque showed characteristics of regression during late time periods (Figure S2B, S2C, S2D and S2E). VCAM-1 expression remained in an elevated state throughout all regression groups although it was not significant in comparison to baseline group (Figure S2A). TGF-β1 mRNA expression returned to their normal levels in subsequent regression phases signifying reduction in local extracellular matrix production.

## Discussion

Atherosclerosis is a focal pathology that occurs as an asymmetric thickening of the arterial *tunica intima* due to a complex array of molecular and cellular events. However, depending on their surrounding *milieu*, atherosclerotic plaques might progress, stabilize or regress [9]. To our knowledge, this is the first study to demonstrate schematic evolution of atherosclerosis in balloon injured rabbit iliac artery by cyclical feeding of atherogenic diet for 8 weeks and subsequent withdrawal up to 64 weeks. The animal model exhibited a biphasic evolution of atherosclerosis which included a brief phase of plaque instability with subsequent regression.

Primarily, microscopic level regression in atherosclerosis includes (1) arrest of intimal cell proliferation; (2) restored integrity of the endothelium lining the plaques; and (3) decrease in the amount of intracellular and interstitial lipid [10]. We characterized these aspects of regression and evaluated key genes involved in atherosclerosis. The first striking feature showed continuous progression of atherosclerotic plaque towards more complexity even after 8 weeks of AD withdrawal. It is well known that development of atherosclerosis in rabbits is mainly lipid driven. The presence of higher plasma TC and LDL-C in Reg 8 week group corroborated the earlier understanding that removal of atherogenic diet in rabbits does not lead to an immediate normocholesteremia. This is due to excess cholesterol accumulation in extravascular sites which later diffuses into the blood thus protracting the hypercholesteremia [40]. During this period, vascular infiltration still occurs which may cause further plaque development [40]. We also observed continuous increase in intima/media thickness ratio and plaque area in Reg 8 week group thus supporting the above fact. Because plaque composition, rather than size, determines the clinical course and consequences of atherosclerosis, the main plaque cellular components, macrophages, MMP-9, SMCs and collagen content were studied. During this period, the lesion progressed to more complexity with increased expression in MMP-9 and ground substance along with maintained macrophage foam cell density. The plaques in this group also exhibited an increased pro-inflammatory cytokine *milieu* as shown by increased mRNA expression of TNF-α, IFN-γ and MCP-1. A corresponding decrease in alpha smooth muscle actin and collagen content was also observed in this group. Hence, the plaques developed in this group fulfilled some criteria of an unstable human atherosclerotic plaque [41]. However, an important difference was the absence of plaque rupture and thrombosis. A notable feature was that lesion areas with enhanced MMP-9 and ground substance showed less collagen content substantiating earlier evidence in humans and animals that dissolution of collagenous matrix is due to over expression of catalytically active MMPs [42]. This may be further ascertained using zymography. It is evident that plaque aging continued even after AD removal for 8 weeks in this model. The development of plaques involves not only structural and compositional changes in the intima, but also synchronous changes in the media. The increased medial breakdown and atrophy along with medial thinning in Reg 8 week group can be attributed to sustained lipid and foamy macrophage content and also due to increase MMP-9 expression [25].

A prominent similarity between rabbit and human lesions is the presence of foam cells [2]. During regression, cholesterol diet removal was accompanied by the disappearance of macrophage foam cells. This was one of the first noticeable indications of plaque regression [43]. One of the mechanisms of macrophage clearance in the early period after cholesterol withdrawal is by apoptosis and phagocytosis by new macrophages [43]. However, defective phagocytic clearance (efferocytosis) of the apoptotic macrophages leads to formation of necrotic cores [44]. However, in this study, there was absence of necrotic core in all the groups. It is also worthwhile to note that HDL levels were normal in late regression groups which rules out the possibility of HDL induced reverse cholesterol transport contributing to plaque regression. Hence, it seems that macrophage clearance might be either by 1) An effective phagocytic clearance and/or 2) regained motility of macrophages within atherosclerotic lesion and migration to regional lymph nodes on improvement of local environment [9]. Recent plaque regression studies in animals, however, have not conclusively addressed these mechanisms. Hence, additional studies with tissue examination at several time points after the cholesterol withdrawal would help in understanding macrophage clearing mechanisms.

Collagen and muscle content in plaques and their reorganization is suggested to play a pivotal role in maintaining plaque stability [45]. Collagen types I and III represent 80%–90% of total collagenous protein in atherosclerotic lesions. Type III collagen is reported to be the major collagen type present in the normal arterial wall, while type I is the predominant collagen in atherosclerotic lesions [45]. The collagen staining of baseline group in this model buttresses this fact (Figure 4). Lipid lowering has been shown to favour collagen accumulation with reduced expression and activity of MMPs [22]. Hence, progressive increase in accumulation of interstitial collagen in the plaque area of late regression groups (Reg 16 week to Reg 64 week) might be due to decreased MMP-9, macrophages and other pro-inflammatory cytokines which permitted the accumulation of arterial extracellular matrix macromolecules such as interstitial collagen. Concomitantly, muscle content in late regression groups were also progressively normalized thus indicating features of a stable plaque.

Blood flow variations may influence the relative residence time of lipoproteins, blood borne molecules and inflammatory cells in various arteries thus altering gene expression profile [8]. Interestingly, VCAM-1 mRNA expression was up regulated consistently during regression phase (Reg 4 week to Reg 64 week). This demands relooking the phenomenon from a newer perspective. Firstly, up regulation of VCAM-1 mRNA expression does not necessarily mean similar protein expression. Secondly, presupposing that VCAM-1 protein expression follows a similar trend, then we may be trying to figure out reparative processes in which VCAM-1 might be playing a role. It is possible that VCAM-1 might be playing a reparative role *via* SMC migration [46] during repairing of medial layer in Reg 64 week group. Thirdly, an artery specific response cannot be ruled out. The lesions evolve actively after AD removal wherein we see increased secretion of TIMP-1 and MMP-9 mRNA expression complex plaque groups *viz*. Reg 4 week and Reg 8 week. The results are in tune with previously published observations in rabbits [47]. From clinical perspective, the results gain importance especially when TIMP-1 and MMP-9 together are recently under intense investigation as a biomarker for stroke and cardiovascular death in humans [21]. Also, potential role of high TIMP-1 expression in arresting intimal cell proliferation in late regression groups cannot be negated [48]. An excess stimulation of mitogens and secretion of the arterial wall components are assumed to contribute to plaque growth and might explain the lack of regression in some species and under certain conditions. Hence, regression in our study may be due to reduction in TGF-β1 expression, a growth factor responsible for extracellular matrix (ECM) production, and reduction in pro-inflammatory cytokines expression (TNF-α, IFN-γ and MCP-1) with simultaneous increase in anti-inflammatory cytokine IL-10. This lends credence to the fact that regression occurred due to change in the local cytokine *milieu* [9]. Lack of availability of rabbit specific antibodies presented a technical hurdle to confirm protein expression of respective cytokines.

Endothelial regeneration is one of the necessitating factors for atherosclerosis regression. In our experimental model, balloon injury is the key triggering and pronounced feature for removal of endothelium. Endothelial cell (EC) injury in this setting is more pronounced and needs a larger area to be regenerated especially in the setting of hyperlipidemia as in our case. The impairment in endothelium dependent relaxation in baseline group may be due to reduced elaboration or increased degradation of endothelium derived NO *via* reduced eNOS expression [31], reduced diffusion of NO from endothelium to vascular intima and media due to intimal thickening [49] and alteration of endothelial cell receptors or second messenger systems preventing eNOS activation by agonist stimulation [50]. Also, loss of NO activity promotes lesion formation and its restoration limits intimal proliferation and promotes regression [51]. It is therefore reasonable to speculate that cholesterol feeding and atherosclerosis impairs the function of the endothelial cell nitric oxide synthase *via* various mechanisms. Results obtained indicate recovery of functional responses of endothelium during plaque regression in Reg 50 week and Reg 64 week groups which might be due to availability of NO *via* increased eNOS expression. This needs to be confirmed in future studies. It is plausible that the migration of arterial ECs from adjacent intact endothelium (from aorta near iliac bifurcation) or bone marrow-derived endothelial precursor cells might be involved in endothelial regeneration [52], [53]. Atherosclerosis progression in this model also led to reduced PE-induced contraction as reflected in baseline group. This is because, atheroma progression into the media causes disruption of the smooth muscle and elastic lamina thus hampering vessel contractility [54]. Also, migration of smooth muscle cells from the media to intima may also contribute to the observed loss of contractile function [54]. Alteration of smooth muscle cell phenotype from the contractile status to the secretory status may also impair vessel contraction [54]. The morphological changes exhibited by respective groups reflect the changes in vascular responses. The recovery of medial layer in late regression groups (Reg 50 week and Reg 64 week) showed simultaneous recovery in contractile responses of the injured arteries.

One needs to appreciate that plaque regression is not simply a rewinding of the sequences of lesion progression, but instead involves mobilization of plaque pathological components *via* alteration of specific pro-atherogenic cellular and molecular pathways. The results support current clinical concepts that long-term plaque reorganization leads to plaque stabilization due to normalized atherogenic conditions. These components appear to be completely reversed in humans who have substantial and sustained lowering of their plasma lipids [55]. It should also be noted that whether reduction in plaque burden is associated with improved outcomes is speculative in humans. However, preliminary data suggests that even small plaque regression may be sufficient to produce clinical benefit [56].

Currently, there is large unmet need for a direct disease modifying drug in atherosclerosis. One of the reasons might be due to lack of complete pathological understanding of atherosclerosis at various artery sites in preclinical models thus resulting in clinical failure of investigative agents. Hence, there is an urgent need to characterize atherosclerotic features at various artery sites in animal models and interpret results from that perspective. Majority of arteries employed for atherosclerosis studies in rabbits are carotid, aorta, iliac and femoral. A head to head comparison of atherosclerosis presentation at these sites under similar experimental conditions will not only help to maintain uniformity in preclinical understanding of artery specific changes but also accelerate atherosclerosis drug discovery.

## Conclusions

**Figure 6:**
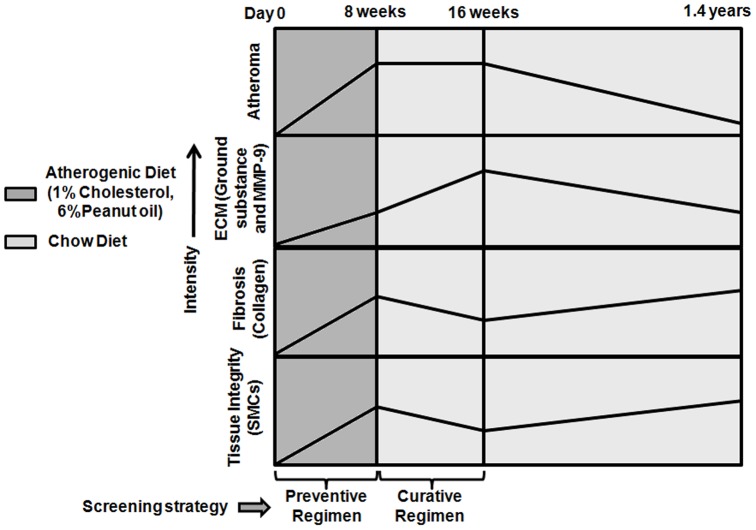
Summary. Schematic evolution of atherosclerosis in balloon injured rabbit iliac artery on atherogenic diet withdrawal over a period of 1.4 years.

The study results indicate that atherosclerotic iliac artery of NZW rabbits presents features of human unstable plaques after a brief period of AD removal. Also, collective changes in plaque intracellular and extracellular lipid, restoration of endothelium functionality, increase in fibrous component (collagen and SMCs), decreased metalloproteinase and modulation of gene expression orchestrated plaque regression associated with this model (Figure 6). The study will not only help researchers to define processes involved in stabilizing or destabilizing plaque structures but also provide the probable window for evaluating new chemical entities on atherosclerosis regression in this model.

## Supporting Information

Figure S1 **Histological examples of morphological changes in rabbit plaques of various groups stained with Movat Pentachrome.** A = IEL duplication at the site of IEL breakage (all groups except Normal), B =  Medial elastic fibres duplication (all groups except Normal), C =  Focal medial breakdown with IEL breakdown (Baseline group section), D = Medial fibrotic reaction (Baseline group section), E =  Lipid core with extracellular lipid and foam cell deposition (Baseline group section), F =  Medial breakdown with no EEL breakage (Reg 8 week section), G = Focal medial compression extending towards atrophy (Reg 8 week section), H = Medial atrophy (Reg 8 week section), I = IEL breakage with no damage to media (Reg 64 week section) and J =  Focal cholesterol clefts (Reg 50 week and Reg 64 week sections). Scale bar = 50 µm.
(TIF)

Figure S2 **mRNA expression (fold change over normal) of key genes involved in atherosclerosis as determined by real time PCR.** (A) VCAM-1 (B) MCP-1 (C) TNF-α (D) IFN-γ (E) TGF-β1 (F) IL-10. *p<0.05 and ***p<0.001 *vs* normal; ^#^p<0.05, ^##^p<0.01 and ^###^p<0.001 *vs* Baseline.
(TIF)

Table S1 **Oligonucleotide primers and specific annealing temperatures for real-time RT-PCR.**
(DOC)
