# Supplementary figures and images for: Cholesterol Diet Withdrawal Leads to an Initial Plaque Instability and Subsequent Regression of Accelerated Iliac Artery Atherosclerosis in Rabbits
Source: PLoS One. 2013 Oct 17;8(10):e77037. doi: 10.1371/journal.pone.0077037 (PMC3798418; doi:10.1371/journal.pone.0077037)

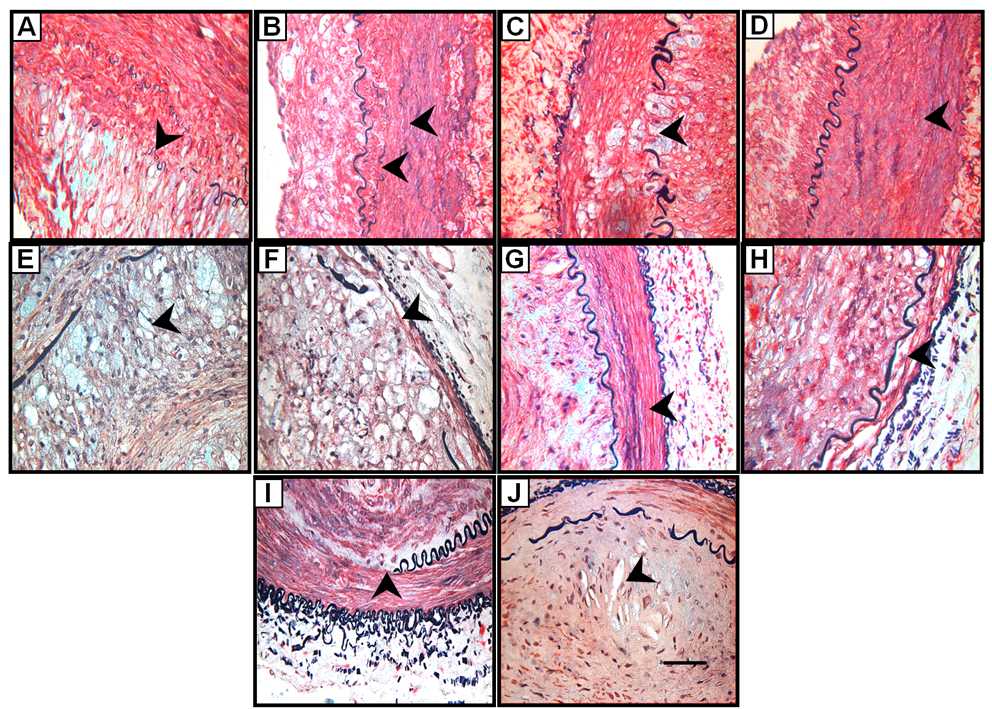

Supplement: Figure S1 — Histological examples of morphological changes in rabbit plaques of various groups stained with Movat Pentachrome. A = IEL duplication at the site of IEL breakage (all groups except Normal), B = Medial elastic fibres duplication (all groups except Normal), C = Focal medial breakdown with IEL breakdown (Baseline group section), D = Medial fibrotic reaction (Baseline group section), E = Lipid core with extracellular lipid and foam cell deposition (Baseline group section), F = Medial breakdown with no EEL breakage (Reg 8 week section), G = Focal medial compression extending towards atrophy (Reg 8 week section), H = Medial atrophy (Reg 8 week section), I = IEL breakage with no damage to media (Reg 64 week section) and J = Focal cholesterol clefts (Reg 50 week and Reg 64 week sections). Scale bar = 50 µm. (TIF) [file pone.0077037.s001.tif]

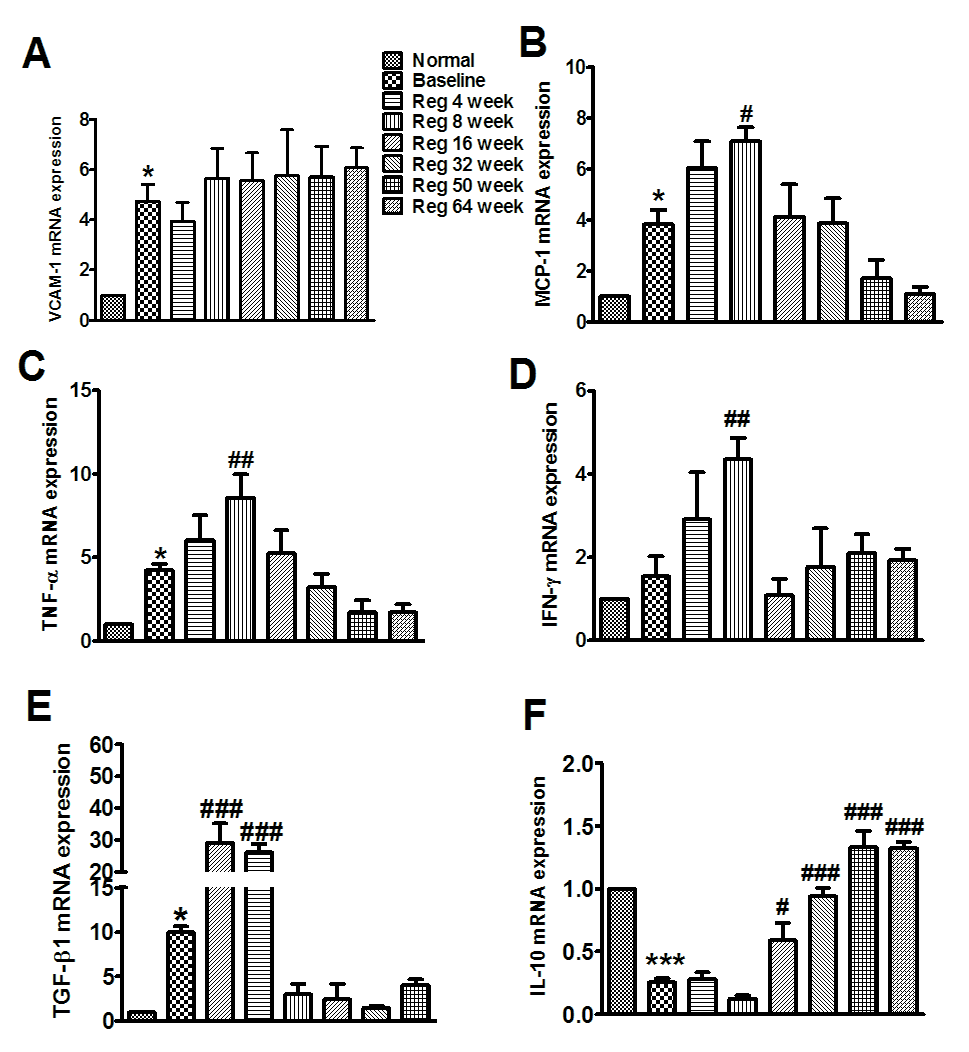

Supplement: Figure S2 — mRNA expression (fold change over normal) of key genes involved in atherosclerosis as determined by real time PCR. (A) VCAM-1 (B) MCP-1 (C) TNF-α (D) IFN-γ (E) TGF-β1 (F) IL-10. *p<0.05 and ***p<0.001 vs normal; #p<0.05, ##p<0.01 and ###p<0.001 vs Baseline. (TIF) [file pone.0077037.s002.tif]
