# Supplementary material for: Cholesterol Diet Withdrawal Leads to an Initial Plaque Instability and Subsequent Regression of Accelerated Iliac Artery Atherosclerosis in Rabbits
Source: PLoS One. 2013 Oct 17;8(10):e77037. doi: 10.1371/journal.pone.0077037 (PMC3798418; doi:10.1371/journal.pone.0077037)
Supplement: Table S1 — Oligonucleotide primers and specific annealing temperatures for real-time RT-PCR. (DOC) [file pone.0077037.s003.doc]

**Table S1. Oligonucleotide primers and specific annealing temperatures for real-time RT-PCR.**

| **Gene** | **Forward primer (5’-3’)** | **Reverse primer (5’-3’)** | **Tm** | **Base pairs** | **Reference** |
| --- | --- | --- | --- | --- | --- |
| **Collagen III** | TTCCTTTTGTTCTAATCTTGTCA | TAGCACCATTGAGACATTTTGA | 60^o^C | 195 |  |
| **Collagen I** | TGAGCCAGCAGATTGAGAAC | CCAGTGTCCATGTCGCAGA | 54^o^C | 143 |  |
| **VCAM-1** | AGTCCCTCGTCCATCGTG | GAAAGAGGCTGTAGGTCC | 60^o^C | 122 |  |
| **TGF-β1** | AGGACGCCAACTTCTGCCT | AGGACCTTGCTGTACTGGGTGT | 60^o^C | 70 |  |
| **IL-10** | GAGAACCACAGTCCAGCCAT | CATGGCTTTGTAGACGCCTT | 60^o^C | 179 |  |
| **MMP-9** | CTTCCAACTTTGACAGCGACA | GGAGTGATCCAAGCCCAGTG | 60^o^C | 110 | NM_001082203.1 |
| **IFN-γ** | CTGGTCCAGCGTAAAGCAGT | TCAGTACTTGGATGCTCGCC | 60^o^C | 126 | NM_001081991.1 |
| **TNF-α** | AGATGGTCACCCTCAGATCAG | GAAGAGAACCTGGGAGTAGATGAG | 60^o^C | 206 |  |
| **MCP-1** | CTTCTGTGCCTGCTGCTCATAG | TGCTTGGGGTCAGCACAGAT | 60^o^C | 221 | NM_001082294.1 |
| **eNOS** | GTGAGACTTTCTGCGTGGGA | CAGACCTGGCAGCAACTGTA | 60^o^C | 131 | NM_001082733.1 |
| **TIMP-1** | AGCAGAGCCTGCACCTGTGT | TGATTGACTTCTGGAGCCCC | 60^o^C | 101 |  |
| **GAPDH** | GAACGGGAAACTCACTGGCAT | CCTTCTTGATGTCGTCATACTTAGC | 54^o^C | 110 |  |
| VCAM: Vascular Cell Adhesion Molecule; TGF: Transforming Growth Factor; IL: Interleukin; MMP: Matrix Metalloproteinase; IFN: Interferon; TNF: Tumor Necrosis Factor; MCP: Monocyte Chemoattractant Protein; eNOS: Endothelial Nitric Oxide Synthase; TIMP: Tissue Inhibitor of Metalloproteinase; GAPDH: Glyceraldehyde 3-Phosphate Dehydrogenase; Tm: Annealing Temperature; RT-PCR: Reverse Transcriptase-Polymerase Chain Reaction | | | | | |

**Supplementary References**:

1. Dong B, Zhang C, Feng JB, Zhao YX, Li SY, et al. (2008) Overexpression of ACE2 enhances plaque stability in a rabbit model of atherosclerosis. Arterioscler Thromb Vasc Biol 28: 1270-1276.

2. Li CJ, Sun HW, Zhu FL, Chen L, Rong YY, et al. (2007) Local adiponectin treatment reduces atherosclerotic plaque size in rabbits. J Endocrinol 193: 137-145.

3. Hofstaetter JG, Wunderlich L, Samuel RE, Saad FA, Choi YH, et al. (2005) Systemic hypoxia alters gene expression levels of structural proteins and growth factors in knee joint cartilage. Biochem Biophys Res Commun 330: 386-394.

4. Godornes C, Leader BT, Molini BJ, Centurion-Lara A, Lukehart SA (2007) Quantitation of rabbit cytokine mRNA by real-time RT-PCR. Cytokine 38: 1-7.

5. Charoenwanthanang P, Lawanprasert S, Phivthong-Ngam L, Piyachaturawat P, Sanvarinda Y, et al. (2011) Effects of Curcuma comosa on the expression of atherosclerosis-related cytokine genes in rabbits fed a high-cholesterol diet. J Ethnopharmacol 134: 608-613.

6. Park KC, Park EJ, Kim ER, Kim Y, Chung SH, et al. (2005) Therapeutic effects of PG201, an ethanol extract from herbs, through cartilage protection on collagenase-induced arthritis in rabbits. Biochem Biophys Res Commun 331: 1469-1477.
